# Supplementary material for: A Comparative Study on Inhibition of Breast Cancer Cells and Tumors in Mice by Carotenoid Extract and Nanoemulsion Prepared from Sweet Potato (Ipomoea batatas L.) Peel
Source: Pharmaceutics. 2022 May 2;14(5):980. doi: 10.3390/pharmaceutics14050980 (PMC9144854; doi:10.3390/pharmaceutics14050980)
Supplement: Supplementary file 1 [file pharmaceutics-14-00980-s001.zip › pharmaceutics-1667444-supplementary.pdf]

Supplementary Materials

for

A Comparative Study on Inhibition of Breast Cancer Cells and Tumors in Mice by Carotenoid Extract and Nanoemulsion Prepared from Sweet Potato (*Ipomoea batatas* L.) Peel

Hsin-Yen Hsu[1] and Bing-Huei Chen[1,2,*]

[1]Department of Food Science, Fu Jen Catholic University, New Taipei City 242, Taiwan; luckypinocat@gmail.com (H.-Y.H).

[2]Department of Nutrition, China Medical University, Taichung 40402, Taiwan.

* Corresponding author (002622@mail.fju.edu.tw (B.-H.C)).

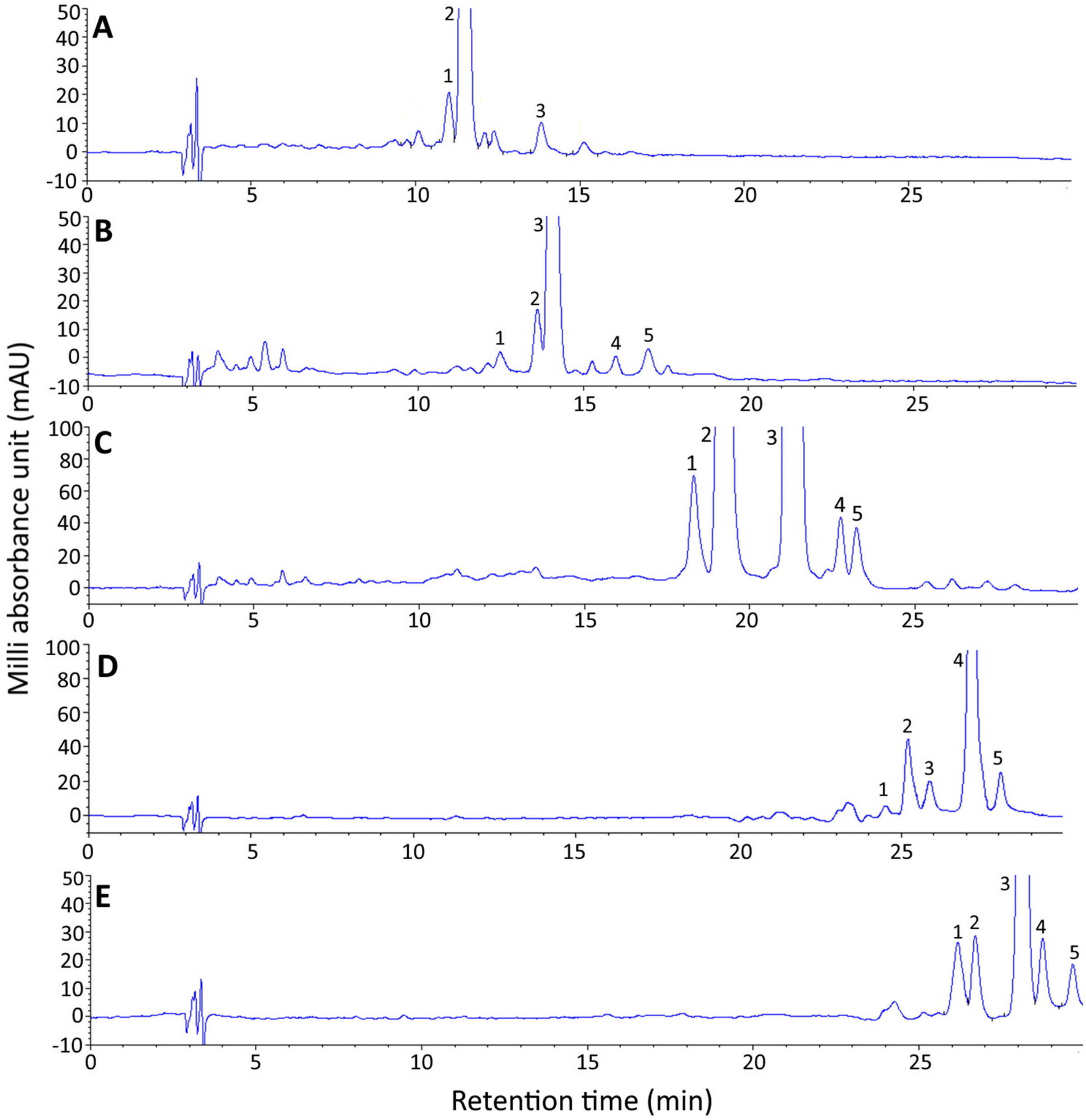

Figure S1 – HPLC chromatogram of all-*trans*-lutein, all-*trans*-zeaxanthin, all-*trans*-β-cryptoxanthin, all-*trans*-β-carotene and all-*trans*-α-carotene standards along with their *cis* isomers during illumination at 25°C for 24 h. A: 1, *cis*-lutein; 2, all-*trans*-lutein; 3, 9- or 9’-*cis*-lutein. B: 1, 13- or 13’-*cis*- zeaxanthin; 2, 15- or 15’-*cis*- zeaxanthin; 3, All-*trans*- zeaxanthin; 4, 9- or 9’-*cis*- zeaxanthin; 5, 9- or 9’-*cis*- zeaxanthin. C: 1, 13- or 13’-*cis*-β-cryptoxanthin; 2, 15- or 15’-*cis*-β-cryptoxanthin; 3, all-*trans*-β-cryptoxanthin; 4, 9- or 9’-*cis*-β-cryptoxanthin; 5, 9- or 9’-*cis*-β-cryptoxanthin; D: 1, 15- or 15’-*cis*-β-carotene; 2, 13- or 13’-*cis*-β-carotene; 3, 9- or 9’-*cis*-β-carotene; 4, All-*trans*-β-carotene; 5, 9- or 9’-*cis*-β-carotene. E: 1, 13- or 13’-*cis*-α-carotene; 2, 13- or 13’-*cis*-α-carotene; 3, All-*trans*-α-carotene; 4, 9- or 9’-*cis*-α-carotene; 5, 9- or 9’-*cis*-α-carotene. The identification data for all the all-*trans* plus *cis* isomers of carotenoids are provided in the Table S2.

Table S1. Identification data for all-*trans* plus *cis* forms of carotenoids in sweet potato peel extract by HPLC-DAD-MS.

Peak no.	Compound	λ (nm, inline)	λ (nm, reported)	Q-ratio found	Q-ratio reported	m/z found	m/z reported
1	All-*trans*-violaxanthin	414, 438, 466	416,439,469 [d]	–	–	601[M+H], 583[M+H-18]	601[M+H], 583[M+H-18] [a]
2	*cis*-lutein	408, 430, 456	336, 412, 432, 456 [a]	0.08	0.12[a]	569[M+H], 551[M+H-18]	569[M+H], 551[M+H-18] [b]
3	All-*trans*-lutein	424, 445, 472	422, 446, 474 [a]	–	0.06[a]	569[M+H], 551[M+H-18], 533[M+H-18-18]	569[M+H], 551[M+H-18], 533[M+H-18-18] [a]
4	All-*trans*-zeaxanthin	430, 454, 476	452, 478 [a]	–	–	569[M+H], 551[M+H-18]	569[M+H], 551[M+H-18] [b]
IS	All-*trans*-canthaxanthin	478	–	–	–	–	–
5	15- or 15'-*cis*-β-cryptoxanthin	338, 423, 448, 474	340,424,446,474 [a]	0.47	0.43[a]	553[M+H]	553[M+H][c]
6	All-*trans*-β-cryptoxanthin	426, 452, 478	428, 450, 477 [b]	–	0.04[b]	553[M+H], 533[M+H-18]	553[M+H], 535[M+H-18] [b]
7	15- or 15'-*cis*-β-carotene	342, 428, 452, 478	342 448 474 [a]	0.43	0.50[a]	537[M+H]	537[M+H] [b]
8	13- or 13'-*cis*-β-carotene	342, 422, 450, 478	345,451,479 [b]	0.32	0.36[b]	537[M+H]	537[M+H] [a]
9	All-*trans*-β-carotene	456, 482	454, 482 [b]	–	–	537[M+H]	537[M+H] [a]
10	All-*trans*-α-carotene	428, 452, 476	424, 450, 476 [c]	–	0.05[c]	537[M+H]	537[M+H] [b]

Based on a report by [a] Kao et al. [11], [b] Strati et al. [17], [c] Kao et al. [18] and [d] Schex et al. [19]. The reference numbers are same as provided in the main text.

Table S2. Identification data for all-*trans* plus *cis* forms of lutein, zeaxanthin, β-cryptoxanthin, β-carotene and α-carotene.

Peak no.	Compound	t_R (min)	*k*	λ (nm, inline)	λ (nm, reported)	Q-ratio found	Q-ratio reported
1	*Cis*-lutein	11.01	2.44	336,420,442,470	332,410,434,458 [a]	0.42	0.31 [c]
2	All-*trans*-lutein	11.48	2.58	336,424,448,476	423, 447, 477 [b]	–	0.04 [b]
3	9- or 9'-*cis*-lutein	13.91	3.34	336,422,444,472	332 423 446 470 [c]	0.10	0.08 [a]
1	13- or 13'-*cis*- zeaxanthin	12.49	2.90	338 424 448 472	338 424 446 472 [d]	0.23	0.37 [b]
2	15- or 15'-*cis*- zeaxanthin	13.68	3.27	340 426 448 472	338 422 446 470 [d]	0.42	0.45 [b]
3	All-*trans*- zeaxanthin	14.16	3.42	456 480	452 478 [a]	–	0.03 [a]
4	9- or 9'-*cis*- zeaxanthin	16.02	4.00	340 426 448 478	338 422 446 474 [d]	0.09	0.12 [b]
5	9- or 9'-*cis*- zeaxanthin	16.98	4.30	340 426 448 478	338 422 446 474 [d]	0.09	0.12 [b]
1	13- or 13'-*cis*-β-cryptoxanthin	18.43	4.75	342 452 472	340 422 446 474 [d]	0.50	0.45 [c]
2	15- or 15'-*cis*-β-cryptoxanthin	19.40	5.06	342 448 472	340 424 446 474 [d]	0.30	0.43 [c]
3	All-*trans*-β-cryptoxanthin	21.38	5.68	454 482	450 477 [b]	–	
4	9- or 9'-*cis*-β-cryptoxanthin	22.85	6.14	342 450 474	336 420 446 473 [e]	0.05	0.10 [a]
5	9- or 9'-*cis*-β-cryptoxanthin	23.42	6.31	342 452 476	336 420 446 473 [e]	0.05	0.10 [a]
1	15- or 15'-*cis*-β-carotene	24.51	6.65	342 458 480	342 448 474 [d]	0.58	0.50 [a]
2	13- or 13'-*cis*-β-carotene	25.24	6.88	344 424 448 474	344 423 446 476 [a]	0.45	0.43 [b]
3	9- or 9'-*cis*-β-carotene	25.88	7.08	344 424 450 476	344, 452, 476 [d]	0.14	0.14 [a]
4	All-*trans*-β-carotene	27.16	7.48	458 484	454 482 [b]	–	–
5	9- or 9'-*cis*-β-carotene	28.18	7.70	344 426 454 478	344, 452, 476 [d]	0.09	0.14 [a]
1	13- or 13'-*cis*-α-carotene	26.21	7.19	334 420 444 470	334 418 442 470 [f]	0.48	0.50 [b]
2	13- or 13'-*cis*-α-carotene	26.73	7.35	336 420 444 470	334 418 442 470 [f]	0.40	0.50 [b]
3	All-*trans*-α-carotene	28.17	7.80	424 452 480	424 450 476 [f]	0.06	0.05 [b]
4	9- or 9'-*cis*-α-carotene	28.73	7.97	340 422 448 474	330 419 441 469 [e]	0.09	0.08 [a]
5	9- or 9'-*cis*-α-carotene	29.60	8.25	340 424 448 474	330 419 441 469 [e]	0.09	0.08 [a]

Based on a report by [a] Inbaraj et al. [12], [b] Straiti et al. [17], [c] Liu et al. [20], [d] Kao et al. [11], [e] Gupta et al. [21] and [f] Kao et al. [18]. The reference numbers are same as provided in the main text.

References

11. Kao, T.H.; Loh, C.H.; Inbaraj, B.S.; Chen, B.H. Determination of carotenoids in *Taraxacum formosanum* by HPLC-DAD-APCI-MS and preparation by column chromatography. *J. Pharm. Biomed. Anal.* **2012**, *66*,144-153.
12. Inbaraj, B.S.; Chien, J.T.; Chen, B.H. Improved high performance liquid chromatographic method for determination of carotenoids in the microalga *Chlorella pyrenoidosa*. *J. Chromatogr. A* **2006**, *1102*, 193-199.
17. Straiti, I.F.; Sinanoglou, V.J.; Kora, L.; Miniadis-Meimaroglou, S.; Oreopoulou, V. Carotenoids from foods of plant, animal and marine origin: an efficient HPLC-DAD separation method. *Foods* **2012**, *1*, 52-65.
18. Kao, T.H.; Chen, C.J.; Chen, B.H. Carotenoid composition in *Rhinacanthus nasutus* (L.) Kurz as determined by HPLC-MS and affected by freeze-drying and hot-air-drying. *Analyst* **2011**, *136*, 3194-3202.
19. Schex, R.; Lieb, V.M.; Jiménez, V.M.; Esquivel, P.; Schweiggert, R.M.; Carle, R.; Steingass, C.B. HPLC-DAD-APCI/ESI-MSn analysis of carotenoid and α-tocopherol in Costa Rican *Acrocomia aculeata* fruits of varying maturity stages. *Food Res Int*. **2018**, *105*, 645-653.
20. Liu, H.L.; Chen, B.H.; Kao, T.H.; Shiau, C.Y. Carotenoids composition in *Scutellaria barbata* D. Don as detected by high performance liquid chromatography-diode array detection-mass spectrometry-atmospheric pressure chemical ionization. *J. Funct. Foods* **2014**, *8*, 605-610.
21. Gupta, P.; Sreelakshmi, T.; Sharma, R. A rapid and sensitive method for determination of carotenoids in plant tissues by high performance liquid chromatography. *Plant Methods* **2015**, *11*, 5.
